# Enhancement of Plasticizing Effect on Bio-Based Polyurethane Acrylate Solid Polymer Electrolyte and Its Properties

**DOI:** 10.3390/polym10101142

**Published:** 2018-10-12

**Authors:** Tuan Syarifah Rossyidah Tuan. Naiwi, Min Min Aung, Azizan Ahmad, Marwah Rayung, Mohd Sukor Su’ait, Nor Azah Yusof, Khine Zar Wynn Lae

**Affiliations:** 1 Department of Chemistry, Faculty of Science, Universiti Putra Malaysia, UPM Serdang 43400, Selangor, Malaysia; tnsyarifahrossyidah@gmail.com (T.S.R.T.N.); azahy@upm.edu.my (N.A.Y.); 2 Higher Education Centre of Excellence (HiCoE), Institute of Tropical Forestry and Forest Products, University Putra Malaysia, UPM Serdang 43400, Selangor, Malaysia; marwahrayung@yahoo.com; 3 School of Chemical Science and Food Technology, Faculty of Science and Technology, Universiti Kebangsaan Malaysia, Bangi 43600, Selangor, Malaysia; azizan@ukm.edu.my; 4 Solar Energy Research Institute (SERI), Universiti Kebangsaan Malaysia, UKM Bangi 43600, Selangor, Malaysia; mohdsukor@ukm.edu.my; 5 Department of Chemistry, Yangon University, Yangon11041, Myanmar; kzwlae@gmail.com

**Keywords:** biopolymer electrolyte, plasticizer, solid polymer, polyurethane acrylate, conductivity

## Abstract

Polyurethane acrylate (PUA) from vegetable oil has been synthesized and prepared for solid polymer electrolyte. Polyol has been end-capped with Toluene 2,4-Diisocyanate (TDI) followed by hydroxylethylmethylacrylate (HEMA) in a urethanation process to produce PUA. The mixtures were cured to make thin polymeric films under UV radiation to produce excellent cured films which exhibit good thermal stability and obtain high ionic conductivity value. 3 to 15 wt. % of ethylene carbonate (EC) mixed with 25 wt. % LiClO_4_ was added to PUA to obtain PUA electrolyte systems. PUA modified with plasticizer EC 9 wt. % achieved the highest conductivity of 7.86 × 10^−4^ S/cm, and relatively improved the linear sweep voltammetry, transference number and dielectric properties. Fourier Transform Infrared Spectroscopy (FTIR) and dielectric analysis were presented. Thermogravimetric analysis (TGA) and differential scanning calorimetry (DSC), followed by X-ray Diffraction (XRD) and morphology have been studied. The addition of plasticizer to the polyurethane acrylate shows significant improvement in terms of the conductivity and performance of the polymer electrolyte.

## 1. Introduction

Polyurethane acrylate (PUA) is a copolymer that consists of urethane linkage (-NHCOO-) and the acrylate group in its molecular structure. It was synthesized from polyol and isocyanate with the addition of acrylate compound [1,2,3,4,5]. Commonly, PUA has been synthesized from polyether or polyester polyol of synthetic polymers as they have reactive sites to react with the isocyanate group. The properties of vegetable oil that can be modified to polyol have been studied to produce bio-based polymer PUA [6,7,8,9,10].

Urethane acrylate is mostly applicable for coating purposes with in situ polymerization via UV and is curable in the presence of reactive diluent and free radical photoinitiator. Urethane acrylate oligomer has been studied specifically for the UV curable polymer. Most of the studies on acrylate polymers are related to the UV radiation preparation method that have very high speeds of curing that lead to high productivity, lower energy consumption, reduction of volatile organic compound emission, reducing risk of fires, improving aspects of occupational safety and health and so forth [11,12]. Moreover, monomers derived from vegetable oil are environmentally friendly and low-cost compared to the synthetic polymer from petroleum.

The enhancement in ionic conductivity of polymer electrolyte can be achieved by the addition of either organic or inorganic filler into electrolyte systems such as TiO_2_, SiO_2_, Al_2_O_3_, ethylene carbonate (EC), and propylene carbonate. [13,14]. Inorganic filler promotes more free lithium ions and produces more amorphous regions in the electrolyte for the transfer of charge carriers. Meanwhile, organic filler acts as a plasticizer to reduce the glass transition temperature, T_g_, of polymer, which helps to increase the segmental motion of the polymer backbone and generate free volume. Therefore, the ions can easily migrate through the void, resulting in a better ionic conductivity [15]. Plasticizers with high electric constant and low viscosity behavior enable them to be incorporated with a polymer host to facilitate the formation of dissociated ions [16]. Incorporation of a plasticizer into a polymer system is also a way to reduce crystallinity and increase the amorphous phase of a polymer electrolyte, thereby increasing the mobility of ions, and generating higher ionic conductivity [17]. The addition of EC increases the degree of salt dissociation and ionic mobility [18].

As the concentration of plasticizer increases, the amorphous nature of the electrolyte increases, thus improving the ionic conductivity of the polymer electrolyte. Plasticizers of poly(ethylene glycol) (PEG) with different molecular weight, PEG_200_, PEG_400_, and PEG_600,_ showed that the conductivity of a polymer electrolyte decreases as molecular weight of plasticizer increases [19]. PEG_200_ has the largest concentration of free ions associated only with the anti-symmetric stretching mode in the matrix polymer. A longer chain length contributes to more oxygen, implying that lithium ions and few ions are available for ionic conduction, hence lower the ionic conductivity in the system. The smooth surface of the polymer electrolyte also observed for polymer with the highest conductivity in the present of EC. P(GMA-co-MMA) matrix has good flexibility of polymer chains with inclusion of EC and consequently improved the ionic conductivity [20].

In this research, biopolymer polyurethane acrylate electrolyte was synthesized from polyol of Jatropha oil in the presence of TDI and HEMA. The effect of plasticizing on conductivity and thermal stability of biopolymer electrolyte were studied. PUA modified with plasticizer EC 9% has the highest conductivity of 7.86 × 10^−4^ S/cm and the dielectric analysis was studied. The synthesized PUA was confirmed by Fourier Transform Infrared Spectroscopy (FTIR). The thermal behavior of the biopolymer electrolyte was studied by thermogravimetric analysis (TGA) and differential scanning calorimetry (DSC) and followed by X-ray Diffraction (XRD) and morphology study.

## 2. Experimental

### 2.1. Material

Jatropha oil was obtained from Biofuel Bionas, Kuala Lumpur, Malaysia and the oil was used as received. Hydrogen peroxide 30% (H_2_O_2_) and *N*,*N*-dimethylformamide (DMF) (98%) were purchased form R&M chemicals, Petaling Jaya, Malaysia. Formic acid (98%) and tolulene 2,4-diisocyanate (TDI) (98%) were purchased from Acros Organic, New Jersey, US). EC, 2-Hydroxyethylmethyl acrylate (HEMA) (99%), 1,6-Hexanediol diacrylate (HDDA), 2-methylpropiopropanone (Darocure) and lithium perchlorate (LiClO_4_) was obtained from Sigma Aldrich, St.Louis, Missouri, USA. All chemicals were used as received without further purification.

### 2.2. Preparation of PUA Oligomer

The PUA oligomer was prepared by reaction of the Jatropha oil-polyol with TDI in a four-necked round-bottom flask equipped with a mechanical stirrer, a thermometer, and a reflux condenser under nitrogen atmosphere. DMF was used as solvent. The TDI was added in a dropwise manner at 60 °C and the reaction was continued for two hours to complete the NCO-terminated pre-polymer after confirmation by FTIR [21]. After that, the reaction was cooled down to 40 °C and HEMA was added in a dropwise manner. After complete adding of HEMA, the temperature was raised to 60 °C and the reaction was continuously stirred for one hour. From time to time, DMF was added to control the viscosity of the mixture since DMF solvent boiling point was high, as was control for usage [21]. In this study, the molar ratio of polyol to TDI and HEMA used is 1:1:1. The resulting PUA was transparent and yellowish in color.

### 2.3. Preparation of PUA Film Electrolyte

**Scheme 1:**
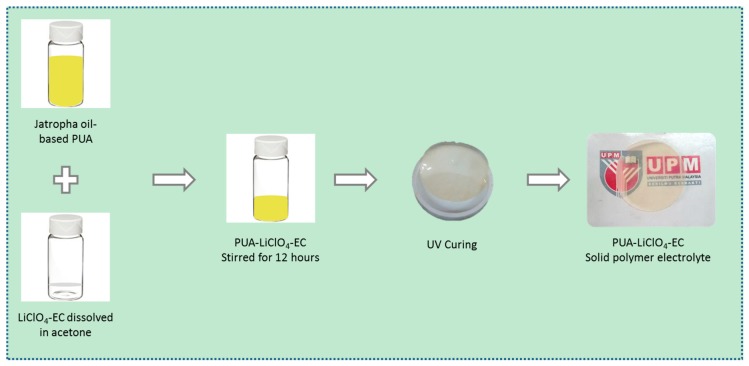
The process of preparing solid polymer electrolyte from PUA and LiClO_4_ and EC.

The PUA solid polymer electrolyte film was prepared by UV-curing and solution-casting method. Initially, the PUA was mixed together with HDDA (monomer) and Darocure (photoinitiator) for 3 h. In separate flask, 25 wt. % of lithium perchlorate salt and EC was dissolved in acetone for 12 h. The amount of EC used was varied from 0–15 wt. % to find the optimum conductivity of the polymer electrolyte [21]. The two mixtures were then mixed together and stirred continuously for another 12 h to obtain a homogenous mixture. After that, the mixture was casted on a Teflon mold (diameter 100 mm) and irradiated 3 times by a medium-pressure mercury vapor lamp from IST-UV Dryer (Switzerland) at 7.5 mA with the conveyor speed at 3 m/s. The solid film shows a uniform and transparent appearance as depicted in Scheme 1. The solid film was stored in a desiccator for one day to remove the residual solvent and undergo further characterization study. The samples were designated as PUA/25Li with 0 wt. %, 3 wt. %, 9 wt. %, 12 wt. % and 15 wt. % EC in all characterizations.

### 2.4. Characterization of Solid Polymer Electrolyte

Infrared spectra were recorded with an ATR-FTIR, Perkin Elmer model 1650 (Waltham, US) in the wavenumber range between 4000 to 280 cm^−1^ with scanning resolution of 4 cm^−1^. TGA was performed using Mettler TA300 (Polaris Pkwy, Columbus Oh, US) from 50 °C to 600 °C with a heating rate of 10 °C/min in a dynamic nitrogen atmosphere at 20 mL/min flow rate. Meanwhile, DSC Mettler TA300 was used to study the phase transition of the polymer electrolyte during the heating process. The study was conducted between −50 °C (for 0% EC), 0 °C (with EC) to 150 °C, at 10 °C scanning rate under nitrogen atmosphere at 10 mL/min flow rate. The structural information was studied by using XRD model D-5000 Siemen (Tempe, AZ, USA). The diffraction angle, 2θ, is in the range of 3 to 60 at 0.004 °/S. Scanning electron microscopy, SEM model JEOL JSM-7600F (Singapore) was used to investigate the surface morphology of the PUA electrolyte. Prior to analysis, the sample was coated with gold to avoid electrostatic charging.

#### Electrochemical Study

The room-temperature ionic conductivity of the PUA SPE was studied by using Electrochemical Impedance Spectroscopy (EIS) using Hioki 3532-50 LCR Hi-tester (Nagano, Japan) that was interfaced to a computer in the frequency range of 50 Hz to 500 kHz. The film was cut into disc shapes with 16 mm diameter and 5.13 cm^2^ contact surface area. The film thickness was 0.05 cm. The film was sandwiched between two stainless steel (SS) electrodes. The ionic conductivity, σ, was calculated based on the following equation:

(1) $$\;\sigma \left({S\;\mathrm{cm}}^{-1}\right)=\frac{l}{A\times R_{b}}\;$$

where *l* is the thickness of the film, *A* is the area and *R_b_* is the bulk resistance obtained from the EIS measurement. The ratio which gave the highest conductivity will be subjected to temperature dependence study from room temperature, 303 to 383 K.

The same ratio was used to study the electrochemical properties of the polymer electrolyte by using Linear Sweep Voltammetry (LSV) and Transference Number (TN) employing Princeton, Versa-STAT-4. For LSV, the sample was assembled in a symmetric SS/PE/Li configuration using 10 mV scan rate in the range of 0–7 V. Sample preparations were carried out in the glove box with less than 0.1 ppm H_2_O and O_2_.

For TN measurement, the PUA electrolyte was assembled in a SS/PE/SS and polarized under a fixed direct current voltage with 0.1 V. The value of *t_ion_* was calculated from the current versus time plot by applying Wagner’s polarization technique following the equation:

(2) $$\;t_{ion}=\frac{I_{o}-I_{ss}}{I_{o}}\;$$

(3) $$\;t_{ele}=\frac{I_{ss}}{I_{o}}\;$$

where *t_ion_* is ionic TN, *t_ele_*—electronic TN, *I*_0_—initial current, *I_ss_*—the steady-state current.

The equations for dielectric constant (*ε_r_*), dielectric loss (*ε_i_*), real electrical modulus (*M_r_*), imaginary electrical modulus (*M_i_*) and tan *σ* were written as below:

(4) $$\;\varepsilon_{r}=\frac{Z_{i}}{\omega C_{o}\left(Z_{r}^{2}+Z_{i}^{2}\right)}\;$$

(5) $$\;\varepsilon_{i}=\frac{Z_{r}}{\omega C_{o}\left(Z_{r}^{2}+Z_{i}^{2}\right)}\;$$

(6) $$\;M_{r}=\frac{\varepsilon_{r}}{\left(\varepsilon_{r}^{2}+\varepsilon_{i}^{2}\right)}\;$$

(7) $$\;M_{i}=\frac{\varepsilon_{i}}{\left(\varepsilon_{r}^{2}+\varepsilon_{i}^{2}\right)}\;$$

(8) $$\;\tan\delta =\frac{\varepsilon_{r}}{\varepsilon_{i}}\;$$

where *C_O_* = *ε_O_A*/*t*, *ε_O_* = permittivity of free space and *ω* = 2πf.

## 3. Results and Discussion

**Figure 1:**
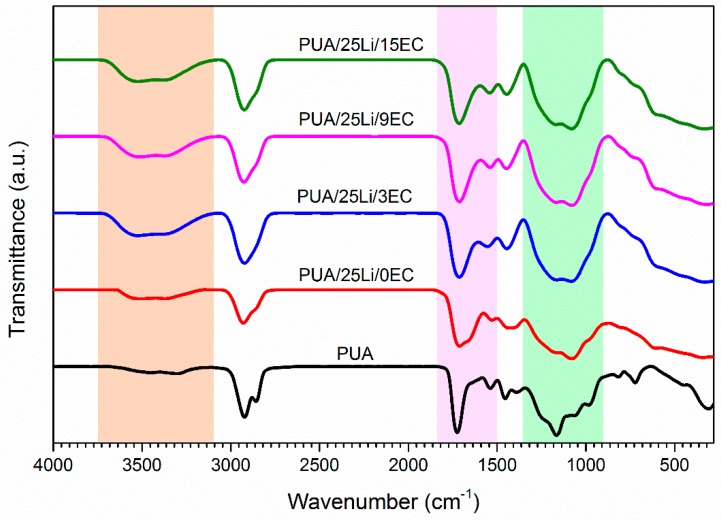
FT-IR spectra of PUA electrolyte at different loading of EC plasticizer.

The FTIR spectra of PUA with 25 wt. % LiClO_4_ in different concentrations of EC are presented in Figure 1. The foremost interest in the PUA polymer electrolytes are region-free and hydrogen bonded –NH stretching mode (3800–3100 cm^−1^), oxygen atoms of the carbonyl (C=O) (1750–1710 cm^−1^, 1650 cm^−1^), amine functional groups (C–N and N–H) (1550–1500 cm^−1^) and ether and ester group (C-O-C) (1300–1000 cm^−1^) stretch of acrylate group [22]. The carbonyl group of first band was electrostatically interacting within the polymer chain and with Li^+^ ions, and the latter is interacting through H-bonds. The absorption peak of free and H-bonded –NH stretching of PUA have shifted (3500 cm^−1^ to 3700 cm^−1^) and showed an increase in the wavenumber when doping with lithium salt due to coordination of Li^+^ ions to nitrogen atoms of the –NH groups. The peak between 1072–1173 cm^−1^ was assigned for the C-O-C stretching band for acrylate group. The urethane groups experienced a shift to a lower frequency (1750 cm^−1^ to 1740 cm^−1^). When adding salt, H-bonded C-O-C groups become free due to an interaction of Li^+^ ions with the ether oxygen and its ability to form H-bonds [23,24]. The PUA region interaction of lithium salt with an addition of EC in the region of oxygen atoms of the carbonyl (C=O) (1750–1710 cm^−1^) and ether and ester group (C-O-C) (1300–1000 cm^−1^) stretch of acrylate group. The peak of polymer electrolyte shows no significant changes of wavenumber in the region of both carbonyl group and ester of stretch acrylate group upon addition of EC. However, an incorporation of EC into polymer electrolyte showed high intensity peak of C=O carbonyl group of PUA. Thus, the plasticizer is essentially a filler dispersed in polymeric composite [16]. With addition of lithium salt the peaks shifted toward higher frequency and the peak of C=O of PUA gave a high-intense, strong, and sharp peak at 1719–1712 cm^−1^. The addition of 25 wt. % lithium shifted the intensity about 7 cm^−1^. This showed an interaction occurred between carbonyl group with lithium salt. The oxygen atoms act as electron donor atoms and form coordinate bonds with the lithium in the structure of polymer host. Thus, vibration frequency shifted to a lower wavenumber.

**Figure 2:**
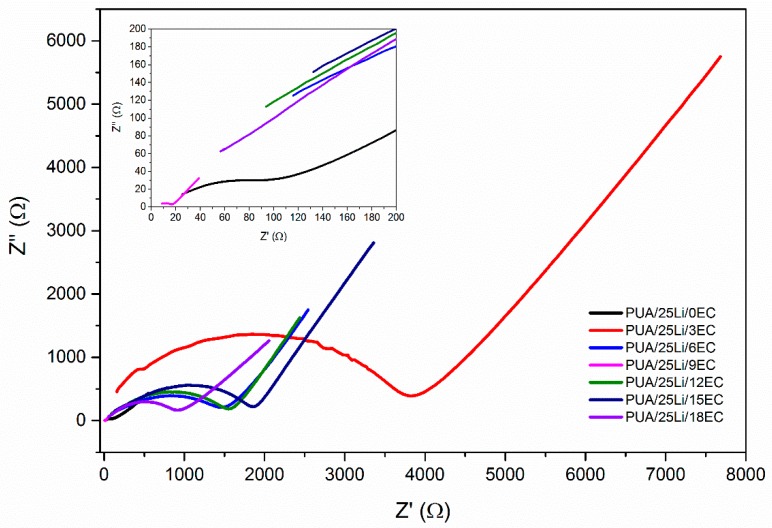
Nyquist plot of PUA electrolyte with EC plasticizer.

**Figure 3:**
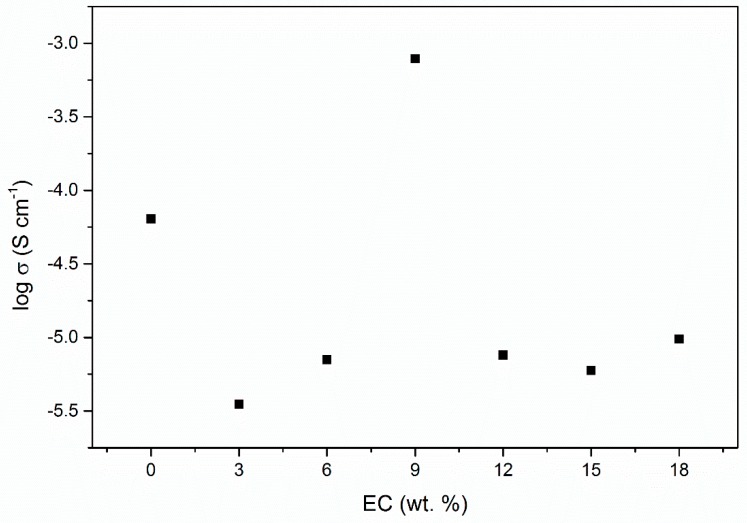
Log conductivity of PUA electrolyte at varied concentrations of EC plasticizer.

The effect that plasticizing has on PUA/LiClO_4_ polymer electrolyte is shown in Figure 2. The spectra shows the bulk resistance for 25 wt. % EC at ~10^−4^ ohm, decreasing to ~10^−5^ at 25 wt. % of EC. A graph of log conductivity versus concentration of EC added in Figure 3 shows the higher conductivity achieved with 9 wt. % of EC and the value is illustrated in Table 1. The addition of EC showed fluctuating value of conductivity and no apparent trend can be observed. However, the optimum conductivity 7.86 × 10^−4^ S/cm had achieved with 9 wt. % of EC, which was one order higher than 0 wt. % EC. The conductivity decreased with addition of 3 to 6 wt. % of EC then the conductivity increased and decreased again at 12 wt. % of EC. EC acts as an organic plasticizer in the polymer electrolyte, enhancing the conductivity of the polymer electrolyte [20]. With the addition of 9 wt. % EC, the conductivity improvement might be due to the high dielectric constant of the plasticizer, increasing the number of ions by weakening the columbic force between anion and cation salts. Therefore, dissociation of salts in the system increased and more free Li^+^ ions can be produced [16]. More Li^+^ ions and EC molecules can transfuse into the polymer matrix and increase the chain segmental mobility, leading to enhancement of conductivity. However, the conductivity starts to decrease, since its saturated level for EC results in less space for Li ions to move within the polymer matrix [20]. The ionic conductivity could be explained by interaction between polymer LiClO_4_ and EC. The main three interactions among them are ion-ion interaction between Li^+^ cations and ClO_4_- anions; ion-dipole interaction between Li^+^ cations and chlorine in polymer; and ion-molecule interaction between Li^+^ and EC. Polymer LiClO_4_-EC with different compounds of polymer –Li^+^, polymer –Li^+^/EC and Li^+^/EC exist by these interactions. The oxygen of C=O in EC is an electron donor which participates in competition with ClO_4_^–^ and polymer. The Li^+^/EC interaction exists not only between Li^+^ and another two oxygen atoms of C=O group, but also between Li^+^ and another two oxygen atoms in the ring structure of EC. Moreover, Li^+^/EC interaction plays an important role in the conductivity of the polymer LiClO_4_-EC system. The addition of EC leads to the formation of a Li^+^/EC complex and enhances the flexibility of polymer chains by decreasing the crystalline fraction of the polymer Li^+^ complex [25].

**Figure 4:**
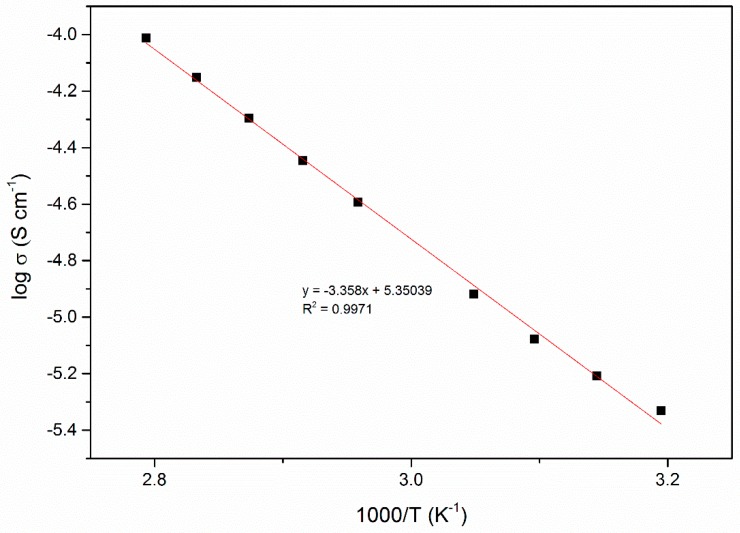
Arrhenius plot of ionic conductivity of PUA/LiClO_4_/EC (9 wt. %) electrolyte.

The conductivity of polymer electrolyte at ambient temperature to 100 °C was fitted using Arrhenius theory. The temperature dependence ionic conductivity was study to analyze mechanism of ion transportation in polymer electrolytes. The log σ versus 1000/T grafted in Figure 4 showed linear plot well-fitted with Arrhenius theory as follows:

σ = A_exp_ [*E*_a_/*kT*] (9)

where A is a constant which is proportional to the amount of charge carriers, *E*_a_ is activation energy, *k* is Boltzmann constant and *T* represents the absolute temperature in K.

**Figure 5:**
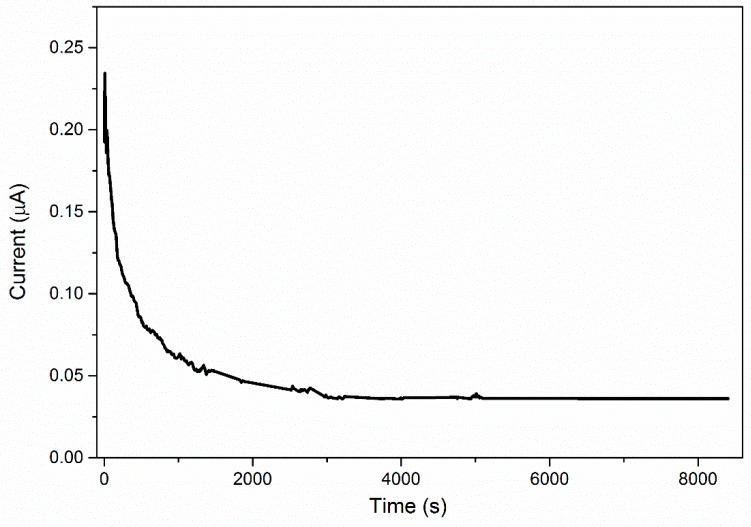
Normalized polarization current versus time for PUA/LiClO_4_/EC (9 wt. %) electrolyte.

**Figure 6:**
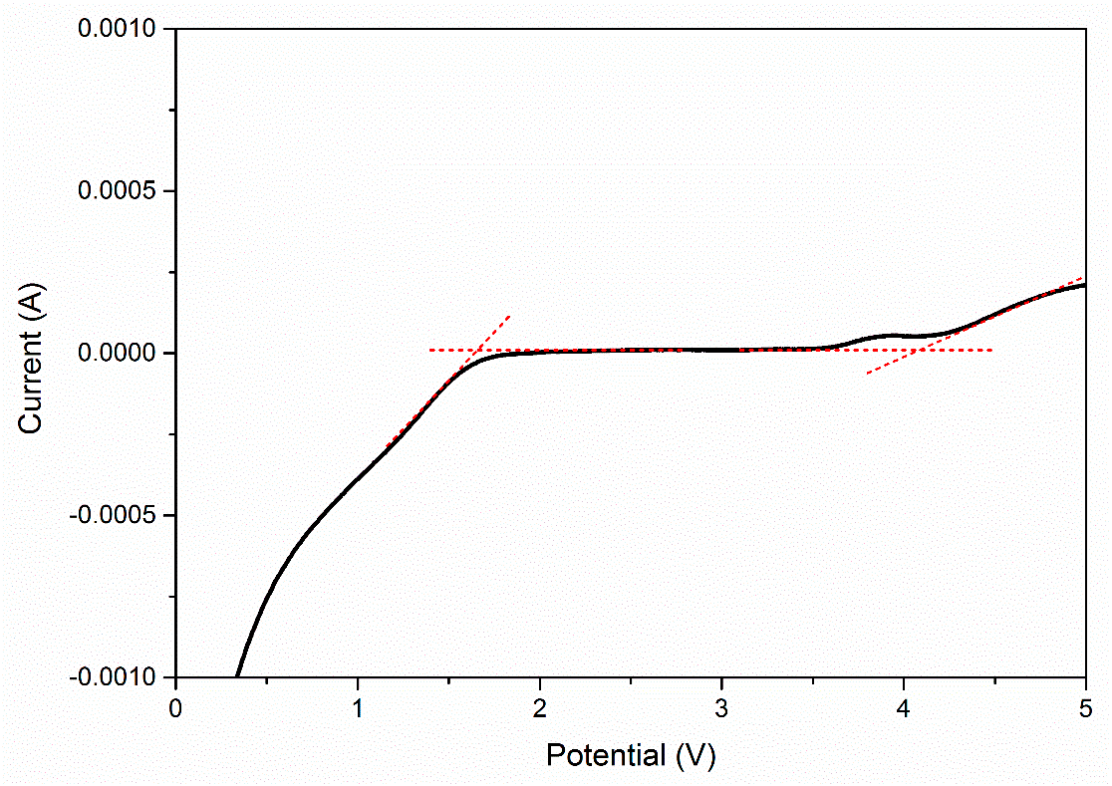
Linear Sweep Voltammetry of PUA/LiClO_4_/EC (9 wt. %) electrolyte.

The pseudo-activation energy, *E*_a_ of the polymer electrolyte was determined from the Arrhenius graph. –*E*_a_/k is denoted on the graph slope and the activation energy is 0.29 eV. The value of activation energy for is related to the ionic conductivity. The higher ionic conductivity only has lower activation energy and vice versa. This indicated that the higher conducting electrolyte requires only a smaller energy to start a migration process [22]. Based on reported study, the lower *E*_a_ provides a smaller band gap which allows the conducting ion to move more easily in a free ion-like state, hence increase ionic conductivity. This phenomenon also related to the conduction process, and lower *E*_a_ is required for the migration of ions in a highly conducting sample. Since migration of ions is tremendously affected by polymer segmental motion, an electrolyte with a lower *E*_a_ facilitates ionic movement, which then increases conductivity [23]. The DC current was observed as a function of time on application of a fixed voltage across SS/polymer electrolyte/SS cells. The normalized current versus time plots was shown in Figure 5 for PUA electrolyte with EC. Based on the plotted graph, the initial total current decreased with time due to depletion of ionic species in the electrolyte and becomes constant in the fully depleted situation. The cell was polarized and current flows when electrons migrate across the electrolyte and interface under a steady-state condition. This is because ionic currents through an ion-blocking electrode fall rapidly with time if the electrolyte is primarily ionic [24]. The value of TN for PUA electrolyte is 0.99, and a PUA electrolyte with plasticizer is 0.95, respectively. This clearly reveals that Li salt complexes with PUA and PUA electrolyte with EC becomes an almost perfect ionic conductor. The reported ionic TN of biopolymer electrolyte containing corn starch by Liew & Ramesh (2013) [26] is 0.98 with ionic conductivity of 3.21 × 10^−4^ S cm^−1^ accepted for a biopolymer electrolyte. The electrochemical stability window of polymer electrolyte was obtained by LSV technique. The sample was sandwiched between SS/PUA electrolyte/Li metal electrodes at ambient temperature. The potential was scanned between 1.0 to 5.0 V at a sweep rate of 5 mV s^−1^. The stability windows are increased from 1.6 to 4.1 V for PUA electrolyte with addition of 9 wt. % EC as plasticizer, as shown in Figure 6. Based on the results, these PUA electrolytes are similar to those of Monisha (2016), which could be used for energy storage devices [27].

**Figure 7:**
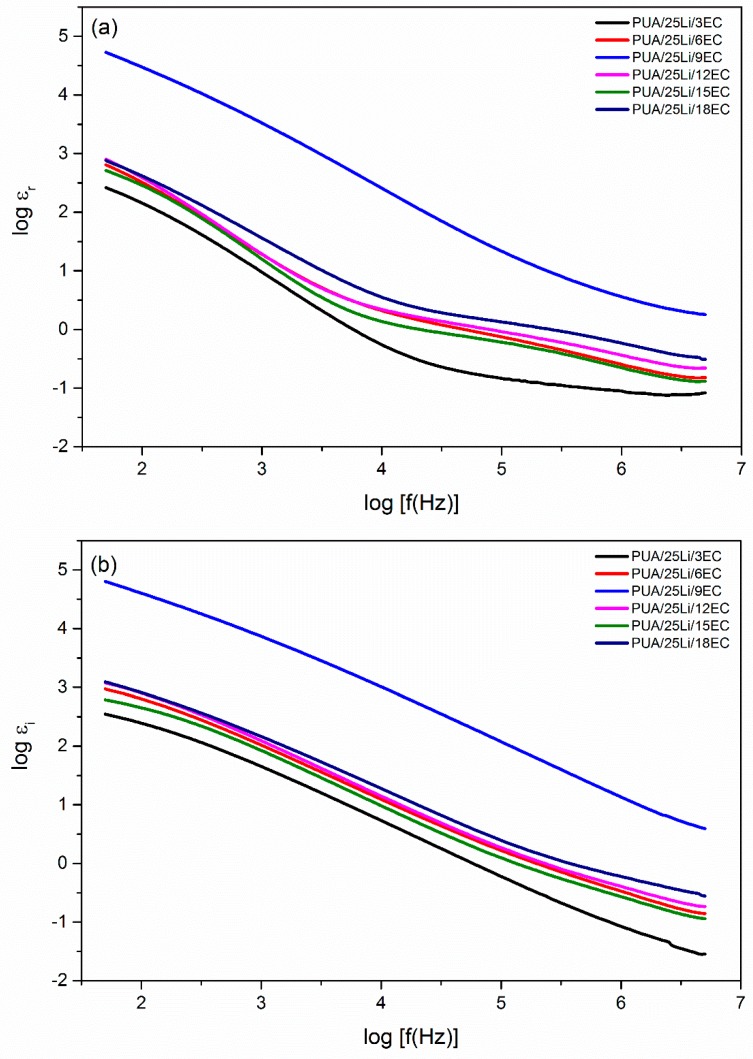
Variation of (**a**) log *ε_r_* and (**b**) log *ε_i_* versus log frequency PUA/LiClO_4_/EC electrolytes.

**Figure 8:**
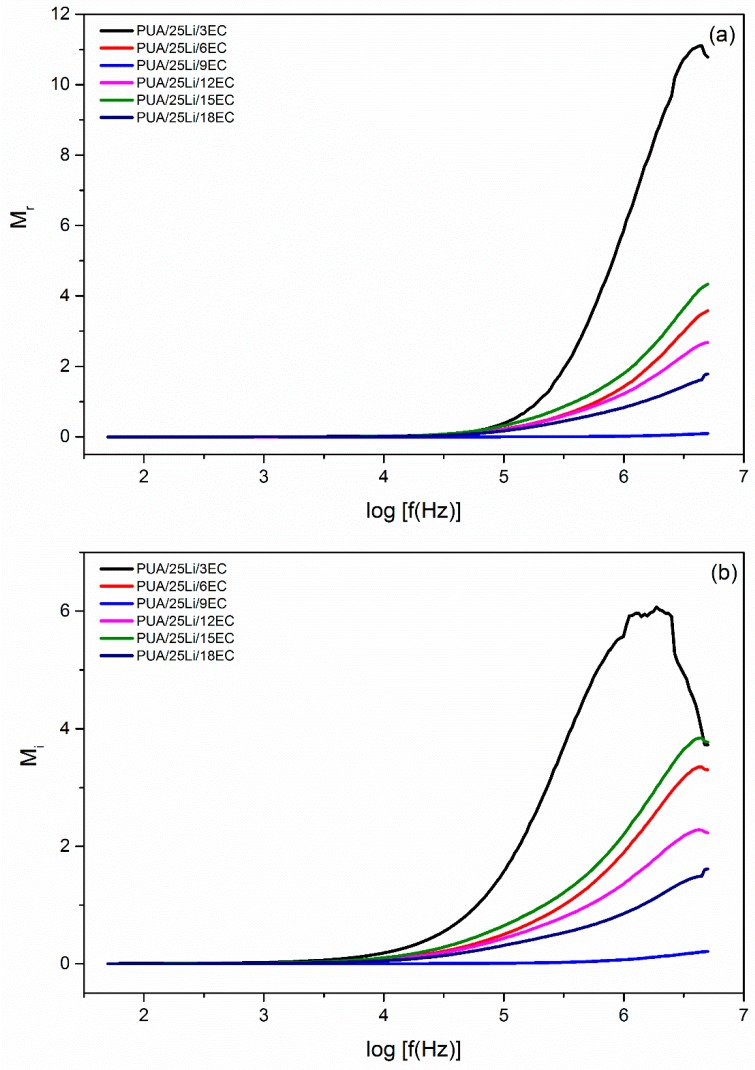
Variation of (**a**) real electrical modulus (*M_r_*) and (**b**) imaginary electrical modulus (*M_i_*) of PUA/LiClO_4_/EC electrolytes.

**Figure 9:**
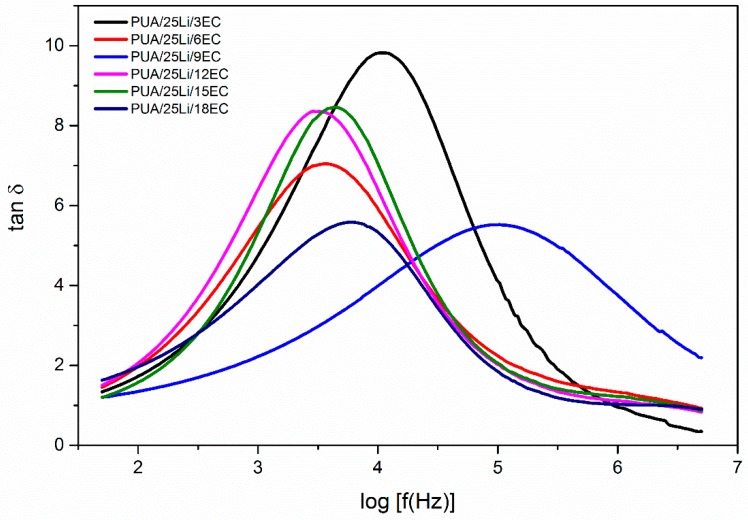
Variation of tan σ with log frequency at different percentage of EC plasticizer.

In this research, EC was added as plasticizer to increase ionic conductivity, σ, in the PUA solid polymer electrolyte. This was because EC (small organic molecules) had high dielectric constant and low vapor pressure [1]. To prove that, log dielectric constant (log *ε_r_*) and log dielectric loss (log *ε_i_*) versus log frequency for different percentages of EC plasticizer with fixed Li content (25% Li) were plotted and shown in Figure 7. From the figure, it can be seen that log *ε_r_* and log *ε_i_* had higher values at low frequency of about 2 Hz. This may be due to electrode polarization effect [28], while at high frequency, it was observed that log *ε_r_* and log *ε_i_* had lower values because of the periodic reversal of electric field occurring at high speed, and this caused the ions to have insufficient time to diffuse into the electric field [28]. Furthermore, with the addition of EC plasticizer, it will cause a large amount of charge carriers to localize along with mobile ions. Thus, it will help to improve the value of ionic conductivity [1]. Figure 8 presented the real electrical modulus (*M_r_*) and imaginary electrical modulus (*M_i_*) with log frequency for different ratio percentages of EC plasticizer added into the fixed Li content. Both *M_r_* and *M_i_* had a value of almost zero with a long tail when approaching high frequency. This happened because of the electrode polarization phenomena, which makes it an insignificant contribution [28]. The presence of the long tail at low frequency (1.5 Hz to 4.5 Hz) observed that there was a large capacitance associated with the electrodes [29]. Other than that, at high frequency, both *M_r_* and *M_i_* observed increased linearity. From Figure 8, it can be seen that *M_r_* peak was much higher than *M_i_* peaks. This was due to the samples of PUA solid polymer electrolyte being an ionic conductor [29]. Figure 6 compared the variation of tan σ with log frequency for different additions of percentages of EC plasticizer into the Li content. From Figure 9, it can be seen that the tan σ peaks were gradually shifted to 3.5 Hz to 5.0 Hz (higher frequency). With increase in EC plasticizer, it can help to increase the amorphous content in the PUA solid polymer electrolyte [27]. As the tan σ peaks shifted toward the right-hand side, it will reduce the relaxation time (τ), which can be demonstrated from Table 2. From Figure 9, PUA 25% Li + 9% EC had the lowest τ which was 0.0955 × 10^−4^ s with highest ionic conductivity up to 7.86 × 10^−4^ S cm^−1^.

**Figure 10:**
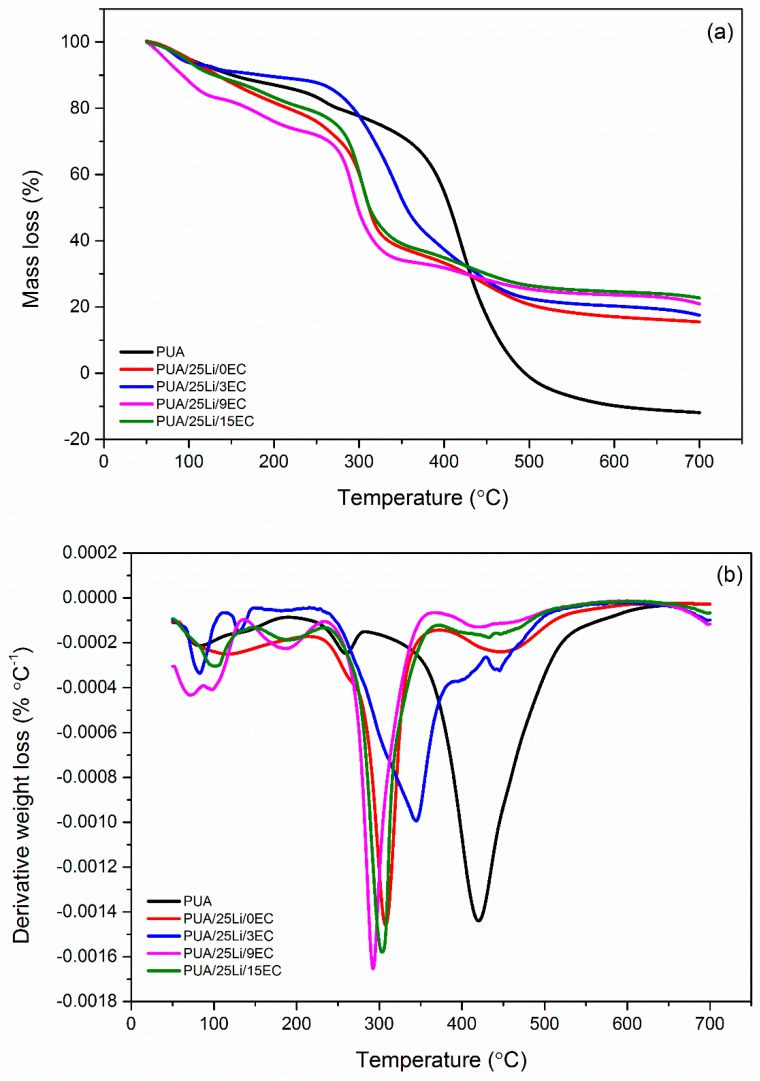
(**a**) TG and (**b**) DTG thermograms of neat PUA and PUA/LiClO_4_/EC electrolytes.

TGA is the preferred technique for evaluation of the thermal stability of a polymer and indicates the decomposition of the polymer at various temperatures. From Figure 10a, the pure PUA has decomposed in three steps beginning at 53.4 °C up to 186.8 °C, followed by a second stage at 199.7 °C to 277.2 °C with T_max_ 253.8 °C. PUA had started to decompose at 53 °C due to decomposition of solvent and moisture about 12.26 wt. % mass loss [30]. There was still a small quantity of solvent degraded at second stage since the mass loss is about 7.43 wt. %, which supports the decomposition of pure PUA at 278 °C with major loss 90 wt. % whenever the pure polymer of PUA has decomposition at single step beginning, as reported by Digar et al. (2002), Santhosh et al. (2006) and Salih et al. (2014) [31,32,33]. From DTG, the major decomposition of PUA at T_max_ 415.8 °C was attributed to the decomposition of organic polymer chains, both the hard segment of urethane linkage and the soft segment from polyether or polyester. The analysis of TG and DTG thermograms was stated in Table 3. From DTG in Figure 10b, the temperature of maximum decomposition, T_max_, for ruptures of urethane linkage decreased upon increased concentration of the salt. The degradation of the PUA hard segment was 415 °C but decreased to 300 °C for PUA 25 wt. % lithium salt. This phenomenon was suggested to be related to the low T_g_ value of polymer electrolyte. The dipole-dipole interaction of polymer chains weakened when lithium salt was added, where it softens the backbone of the polymer chain and reduces the T_g_ of the polymer. The same pattern result was obtained by Ugur et al., 2013 [34]. Based on Figure 10a,b, the decomposition of PUA electrolyte with EC was started at 50 °C due to the loss of moisture and solvent at the first stage of decomposition with 6–20% weight loss. There were three decomposition stages of polymer, as evaluated in Table 3. The major weight loss of polymer with T_max_ 250–350 °C was decomposition of urethane linkage, as mention previously. As reported by Ramesh & Ling, 2010 [35], the increase of plasticizer reduces the thermal stability of the polymer matrix. In this case, the polymers with EC have lower decomposition temperature compared to the without one.

**Figure 11:**
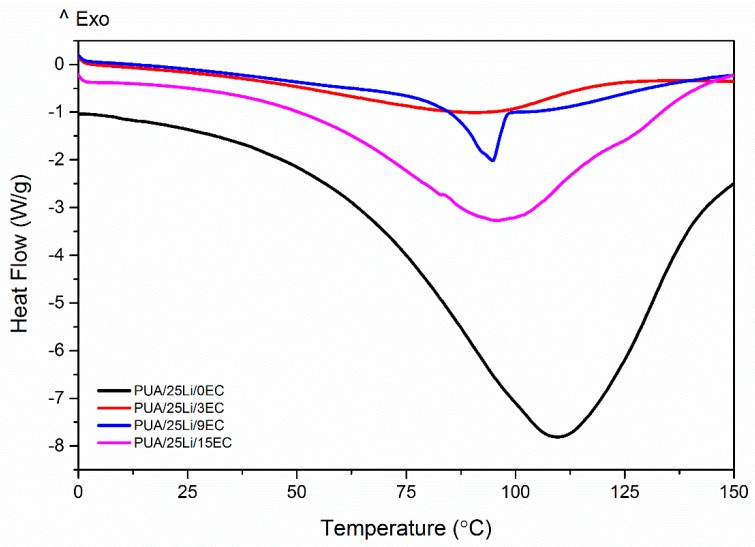
DSC thermograms PUA/LiClO_4_/EC electrolytes.

The thermal behavior of polymer electrolyte with difference concentration of EC was obtained by DSC thermogram at Figure 11 and illustrated in Table 4. Figure 11 showed that the PUA sample only exhibited the glass transition temperature measured at −18.1 °C showed similar result with Feng et al. (2012) [36]. Each samples of polymer electrolyte showed one T_g_ with addition of Li salt. Digar et al. suggest three major types of interaction of polymer electrolyte. (1) interaction of the ether oxygen with the Li^+^ ions, leading to the formation of transient crosslinks between the polyether chains via the Li^+^ ions, which restricts the segmental motion; (2) interaction of urethane –NH and carbonyl groups with the Li^+^ ions leading to inter or intra molecular crosslinking; and (3) mixed ether-urethane interactions with the Li^+^ ions leading to phase mixing of the hard and soft segments. Oxygen present in the acrylate group may also coordinate with Li^+^ ions [16]. The interaction of Li^+^ happened within ether oxygen and possibility of interaction with oxygen present in acrylate group increased the T_g_ of hard segment. This suggests that addition of salt transforms PUA into amorphous.

**Figure 12:**
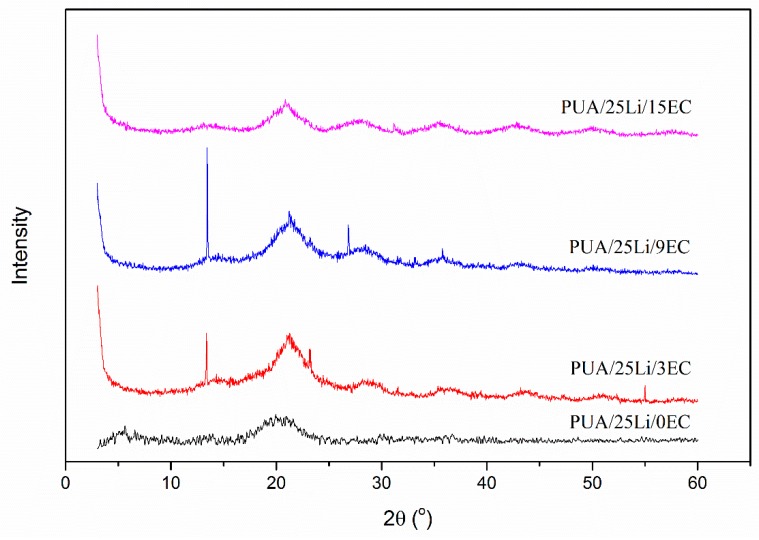
XRD pattern of PUA/LiClO_4_ electrolyte at different EC concentrations.

XRD analysis was carried out to study the crystallinity behavior of the polymer materials. Figure 12 shows the XRD pattern of PUA and PUA with EC electrolyte. The addition of EC to the PUA decreases the semi-crystalline phase by reducing the hump to a broad shape in the region between 15° to 25°. By referring to the XRD pattern of LiClO_4_ as reported by [37], the semi-crystalline peaks are peaks of lithium salt. The lithium salt may be associated from the polymer matrix as shown in the SEM discussion, and agglomeration of polymer exists. PUA with EC has four broader humps of the amorphous region. The amorphous properties of polymer give significant reason for the increment of ionic conductivity, which might be due to the free volume created by continuous segmental motion of polymer chain. This helps ion migration and facilitates the movement of ions [38]. The amorphous nature of the polymer electrolyte was responsible for reducing T_g_ value. It was shown that the more amorphous polymer had low T_g_. This explains that the low T_g_ in the amorphous phase had caused the polymer chains to produce faster bond rotations and segmental motion, hence giving better ionic mobility to the polymer electrolyte. The more amorphous region of polymer leads to a more disordered arrangement of the polymer matrix. Hence, the disorder induces more flexible polymer backbone then increases the mobility of charge carriers [20]. The PUA electrolyte with plasticizer shows good ionic conductivity.

**Figure 13:**
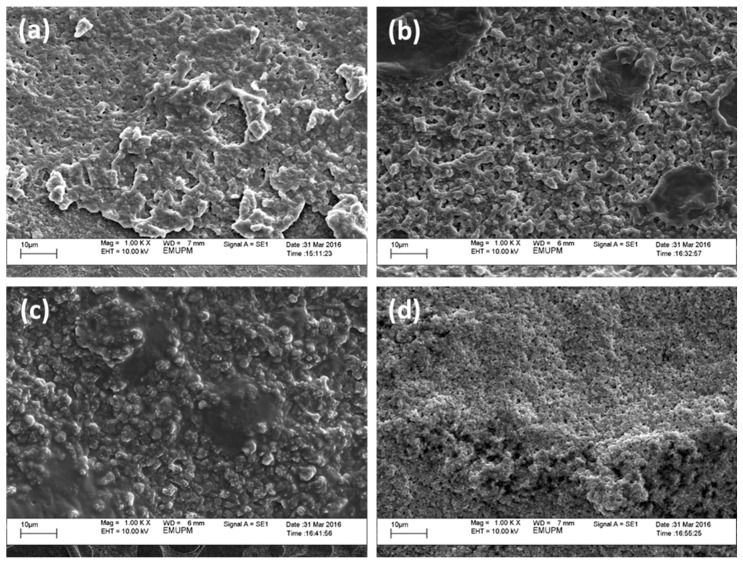
SEM micrograph PUA/LiClO_4_ 25% electrolyte with EC (**a**) 0% (**b**) 3% (**c**) 9% (**d**) 15%.

The SEM micrograph for PUA electrolyte with EC 3 wt. %, 9 wt. % and 15 wt. % is shown in Figure 13a–d. The surface morphology of PUA electrolyte is rough and porous surface. The surface roughness increased with addition of EC. Incorporation of EC with 3 wt. % and 15 wt. % have dark spot (porous structure) assumed phase separation occurred between EC and polymer matrix. Based on Ulaganathan et al. [37], this porosity leads to the entrapment of large volumes of liquid in the pores enhancing higher ionic conductivity. Pores present when the solvent evaporates then increase the amorphous region and solvent retention ability in the electrolyte system. The 9 wt. % EC seems to be optimum for this system as Figure 13c shows smoothest surface morphology and no phase separation occurred. The smoother surface enhances the conductivity because the conducting ions will move freely in the electrolyte. The 9 wt. % EC has higher conductivity due to increase of the ions in the polymer matrix [16]. Furthermore, the crystallinity of lithium salt presence leads to higher ionic conductivity. The spherical sizes relate to the lithium salt in the polymer matrix so that lithium does not dissolve completely on the polymer matrix. However, with the addition of 15 wt. % EC, the dark spot and rough surface morphology resulted in low ionic conductivity compared to 9 wt. % EC.

## 4. Conclusions

The solid polymer electrolytes of PUA from polyol of Jatropha oil with different concentrations of EC have been successfully prepared by solution-casting method under UV irradiation technique. The highest conductivity of PUA electrolyte at room temperature was achieved approximately ~7.86 × 10^−4^ S/cm at 9 wt. % EC with 25 wt. % LiClO_4_ which is increased by five magnitudes of order from the pure PUA. Interaction of lithium ions with oxygen atoms at ether group was shown in infrared spectra. Incorporation of EC into polymer electrolyte was shown in the high-intense of peak of the C=O carbonyl group of PUA. Amorphous behavior of the polymer electrolyte can be observed by morphology study and was then confirmed by the thermal behavior of polymer electrolyte of TGA and DSC. XRD also showed the polymer electrolytes are amorphous in the presence of lithium salt by absence of crystallinity peak. Therefore, the ideal material for practical application with a wide range, battery and solar cell application could show potential useful application.

## Figures and Tables

**Table 1:** Ionic conductivity of PUA electrolyte with EC plasticizer.

EC, wt. %	Conductivity, σ (S cm^−1^)
0%	6.40 × 10^−5^
3%	3.51 × 10^−6^
6%	7.05 × 10^−6^
9%	7.86 × 10^−4^
12%	7.59 × 10^−6^
15%	5.95 × 10^−6^

**Table 2:** Relaxation parameter for different ratio percentage of EC plasticizer.

EC Content (%)	Maximum Peak (Hz)	τ (×10^−4^ s)
3	4.04	0.912
6	3.56	2.75
9	5.02	0.0955
12	3.46	3.47
15	3.61	2.45
18	3.78	1.66

**Table 3:** Analysis of TG and DTG of PUA and PUA/LiClO_4_/EC electrolytes.

Composition	Decomposition Stage	Onset	Final	Temp of Max Decomposition (°C)	Weight Loss (%)	Residue (%)
		Degradation Temp (°C)				
PUA 0 wt. % Li	d_1_	53.38	186.75	79.43	12.26	0
	d_2_	199.67	277.18	253.75	7.43	
	d_3_	278.02	646.83	415.77	90.45	
0 wt. % EC	d_1_	55.98	208.62	107.07	19.62	17.34
	d_2_	209.87	371.83	304.55	44.59	
	d_3_	372.66	579.84	447.94	18.1	
3 wt. % EC	d_1_	53.67	108.96	82.88	6.58	21.14
	d_2_	112.41	146.72	129.37	1.99	
	d_3_	215.72	544.02	345.07	67.93	
9 wt. % EC	d_1_	52.12	132.48	97.77	16.92	23.76
	d_2_	134.24	233.3	185.55	9.94	
	d_3_	234.36	363.81	294.33	39.45	
	d_4_	368.36	585.98	424.8	9.57	
15 wt. % EC	d_1_	54.7	141.67	97.51	11.03	18.46
	d_2_	146.95	230.59	187.2	8.12	
	d_3_	234.49	368.37	305.06	42.57	
	d_4_	353.33	520.54	433.31	11.09	

* All °C values are ±0.05 °C deviation.

**Table 4:** Tm of PUA electrolyte with EC.

Sample	T_m1_ (°C)	T_m2_ (°C)	T_g_ (°C)
PUA 0 wt. % EC	108.9		−15.7
PUA 3 wt. % EC	88.94		
PUA 9 wt. % EC	94.74	156.50	
PUA 15 wt. % EC	95.89		

T_m_—melting temperature; T_g_—glass transition temperature.
